# Probing atherosclerotic angiogenesis with new manganese-based nanocolloid for T1-weighted MRI

**DOI:** 10.1186/1532-429X-14-S1-O11

**Published:** 2012-02-01

**Authors:** Kezheng Wang, Dipanjan Pan, Anne H Schmieder, Huiying Zhang, Angana SenPan, Todd A Williams, Grace Hu, Shelton D Caruthers, Samuel A Wickline, Baozhong Shen, Gregory Lanza

**Affiliations:** 1 Center For Translational Research in Advanced Imaging and Nanomedicine, Cardiovascular Division, Washington University School of Medicine in St.Louis, St.Louis, MO, USA; 2 Molecular Imaging Center and Radiology Department, 4th Affiliated Hospital of Harbin Medical University, Harbin, China

## Summary

This study describes a novel T1-weighted MR molecular imaging approach for sparse epitopes, such as the αvβ3-integrin receptor in atherosclerotic angiogenesis, utilizing a soft Mn-based nanoparticle with high-relaxivity augmented by minute amounts of surface gadolinium. This new agent utilizes about 1/30th the amount of lanthanide used for PFC paramagnetic particles and reduces gadolinium exposure by 300-fold compared with clinical single dose Gd-DTPA.

## Background

We have previously reported in hyperlipidemic rabbit models that integrin-targeted gadolinium perfluorocarbon nanoparticles can effectively assess plaque angiogenesis using MRI. However, the concern for Nephrogenic Systemic Fibrosis (NSF) has motivated the development of alternative technologies that dramatically lower Gd use. We have developed a manganese oleate based nanoparticle with high longitudinal relaxivity for fibrin-specific imaging of thrombus. However, for sparse receptors, such as neovascular integrins, preliminary studies revealed that paramagnetic nanoparticles with greater T1w effects were needed. The objective of this research was to evaluate a manganese-gadolinium nanocolloid (MnGd NC) with gadolinium levels of less than 300X current clinical dosage levels, while providing high levels of T1-weighted MR imaging of sparse angiogenic αvβ3-integrin expression in vivo.

## Methods

A new nanocolloid comprised of a bivalent manganese oleate (0.49±0.02 mg Mn/ml) /polysorbate core encapsulated with phospholipid surfactant enriched with 1.25 mole% Gd-DOTA-cholesterol and 0.3 mole% of quinolone-derived peptidomimetic αvβ3-integrin antagonist-coupled to phosphatidylethanolamine through a PEG(2000) spacer was produced (Diam., 134±2 nm.; Zeta, -25±02mv; PDI, 0.13±0.03). Hyperlipidemic New Zealand White rabbits (n=8), fed a 0.25% cholesterol diet (egg-derived) for 12 months, received αvβ3-integrin-MnGd NC or nontargeted MnGd NC; αvβ3-Integrin-MnGd NC was also administered to rabbits fed a normal chow (n=4). Dynamic 3T MR imaging of the descending thoracic aorta was performed over 2 hours post-injection. Molecular imaging results were corroborated microscopically using fluoresence microscopy.

## Results

MR signal enhancement at 2 hours was increased 18.7±1.95% when averaged over all slices and voxels of the aortic wall in hyperlipidemic rabbits treated with αvβ3-integrin-MnGd NC. (Fig) Signal enhancement due to nonspecific neovascular accumulation of nontargeted MnGd NC was significantly less, (2.5±1.17%, p<0.05). Rabbits fed a normal diet and treated with αvβ3-integrin-MnGd NC had the signal increase, 3.17±1.94%, which did not differ from the nontargeted control. The MRI pattern observed was spatially heterogeneous along both transverse and longitudinal planes of the descending aorta, and predominantly localized between the left subclavian and the diaphragm. Immunohistochemistry corroborated the prominent, neointimal proliferation among cholesterol-fed, atherosclerotic rabbits and the sparse incidence of neovasculature in the control animals. Fluorescently labeled αvβ3-integrin-MnGd NC was localized to the advential neovessels of the lipid rich plaques.

## Conclusions

These data describe a novel T1-weighted MR molecular imaging approach to detect sparse epitopes, such as the αvβ3-integrin receptor in atherosclerotic angiogenesis, which reduces the use of gadolinium by 300-fold of that used clinically in a single dose Gd-DTPA contrast study.

## Funding

We acknowledge grant support from the National Institutes of Health (R01CA154737-01A1, R01 HL094470, R01NS059302, UO1NS073457 for GML and R01073646 for SAW), US Army Medical Research Acquisition (GML) , the International Cooperation and Exchanges Program of the National Ministry of Science and Technology (2009DFB30040) (for BS), the National Natural Science Foundation of China (30970807 and 30570527) (for BS), the National Natural Science Foundation of China for Young Scholar (81101086) (for KW), China Postdoctoral Science Foundation (20100471020) (for KW) and Medical Scientific Research Foundation of Heilongjiang Province health department (2010-156) (for KW).

